# Supplementary material for: Linking aerobic scope to fitness in the wild reveals potential opportunities to help recover imperiled salmon populations
Source: Commun Biol. 2026 Feb 4;9:359. doi: 10.1038/s42003-026-09642-7 (PMC12979789; doi:10.1038/s42003-026-09642-7)
Supplement: Supplementary file 1 — Supplemental material [file 42003_2026_9642_MOESM1_ESM.pdf]

Supplementary information for:

Linking aerobic scope to fitness in the wild reveals potential opportunities to help recover imperiled salmon populations

This file includes:

Supplementary Figures S1-S18
Supplementary Tables S1-S5

Other supplementary information for this manuscript includes:

DataS1_R1.csv, DataS2.csv, DataS3.csv, DataS4.csv, DataS5.csv, DataS6.csv, DataS7_R1.csv, Code S1_R2.R, CodeS2_R2.R, and CodeS3_R2.R

These files are described below, and can be downloaded using the following link: https://doi.org/10.5061/dryad.kprr4xhdr

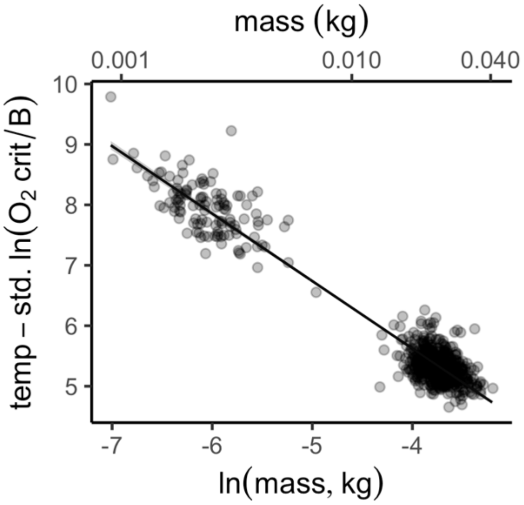

Supplementary Figure 1

Linear regression (LR) associating the natural logarithm of temperature-standardized, mass (B)-specific O_2crit with the natural logarithm of mass (kg) for juvenile Chinook salmon. Points show data, while the line and ribbon respectively show the regression fit and 95% CI (ribbon is very small). Note that this fit was purely to mass-standardize the data and should therefore be interpreted within the bounds of this objective. The secondary x-axis (above) shows the corresponding mass in kg.

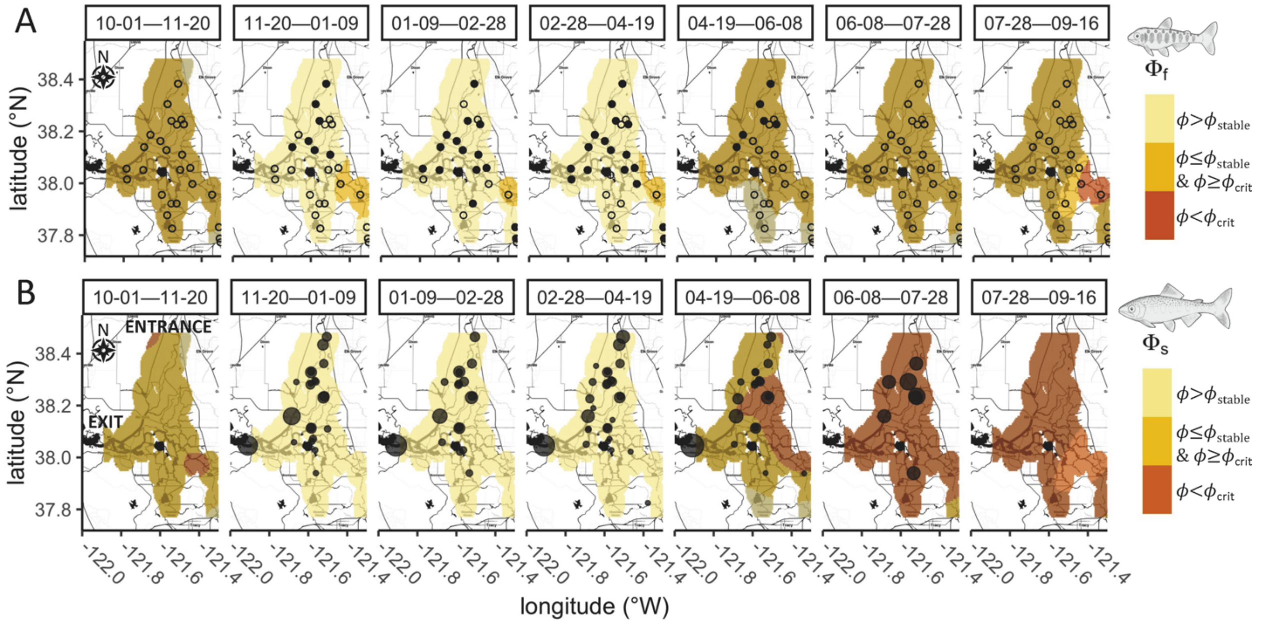

Supplementary Figure 2

Predicted spatial patterns of ϕ for (A) fry and (B) smolts averaged over 50-day date bins. ϕ_f=fry-specific ϕ, and ϕ_s=smolt-specific ϕ. If present, greyed regions indicate above-average predation probability by largemouth bass (see Figure 5). Points plotted in (A) and (B) show juvenile Chinook salmon data collected within the corresponding 50-day bins over 2019-2021 water years. In (A), empty points show locations where sampling efforts caught no fry (i.e., habitat not used for rearing), while filled points show locations where fry were caught (i.e., habitat used for rearing). In (B), points show the location of last known detections (i.e., unsuccessful migration), sized by the proportion of smolts detected during the day of water year bin. The entrance and exit to the delta are marked in the first panel (10-01—11-20); points at the exit show fish that were subsequently detected further downstream (i.e., successful migration). The spatial pattern of ϕ was seasonally consistent for both lifestages: declines in ϕ began in the interior Delta in winter and spread northwest and upstream through fall; by the following winter, ϕ had recovered in all regions but the interior Delta. The spatial patterns of (A) rearing and (B) migration generally co-occurred with changes in ϕ: rearing fry began to utilize Delta habitats as $\phi>\phi_{stable}$, and their detection in surveys mostly ceased after $\phi\leq\phi_{stable}$. The exception was the Sacramento River (Supplementary Figure 10), where fry continued to be detected while ϕ was at and between ϕ_{stable} and ϕ_{crit} (04-19—06-08), highlighting the importance of this region for rearing. Smolt migration attempts occurred as $\phi>\phi_{stable}$ Delta-wide, and some migration attempts continued to be successful even after $\phi\leq\phi_{stable}$ along the major migratory routes (Supplementary Figure 10). However, once $\phi<\phi_{crit}$, successful passage ceased along all routes. If present, above-average predation probability generally occurred delta-wide and on the fringes of juvenile Chinook salmon presence—the respective exceptions being the southeast Delta during summer and early fall (07-28—09-16), when ϕ may have been too low for bass to attempt digestion, and smolt migration in spring and summer (06-08—07-28), the latter of which was never successful.

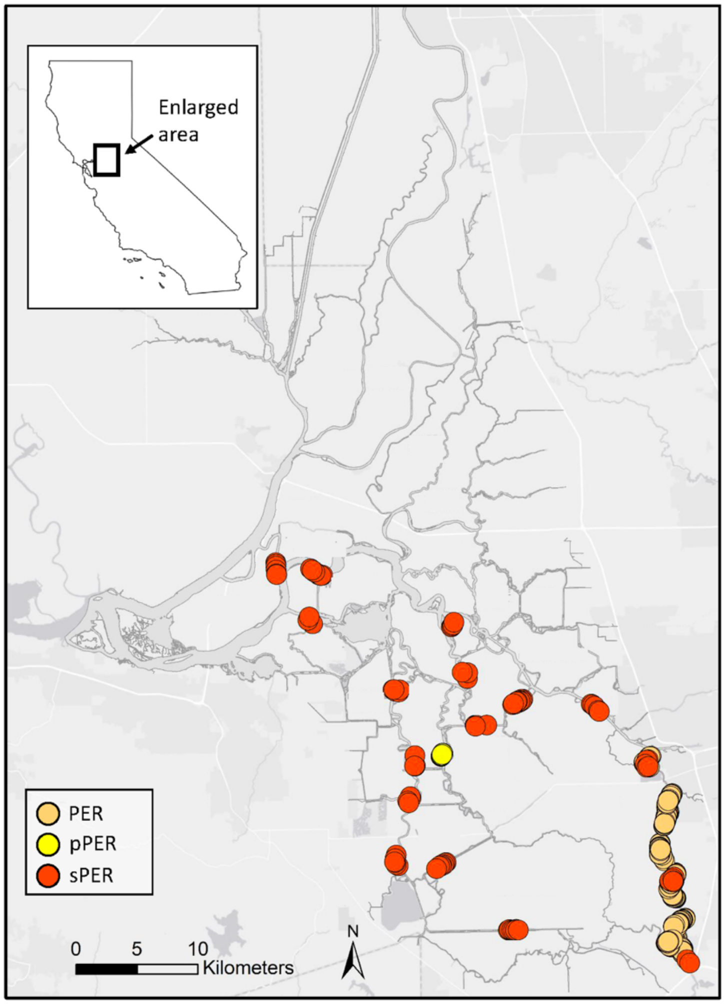

Supplementary Figure 3

Map of the Delta study area overlaid with locations of Predation Event Recorder (PER) deployments (sPER=stationary PER, pPER=pole PER). Points show the GPS location at the midpoint of deployments.

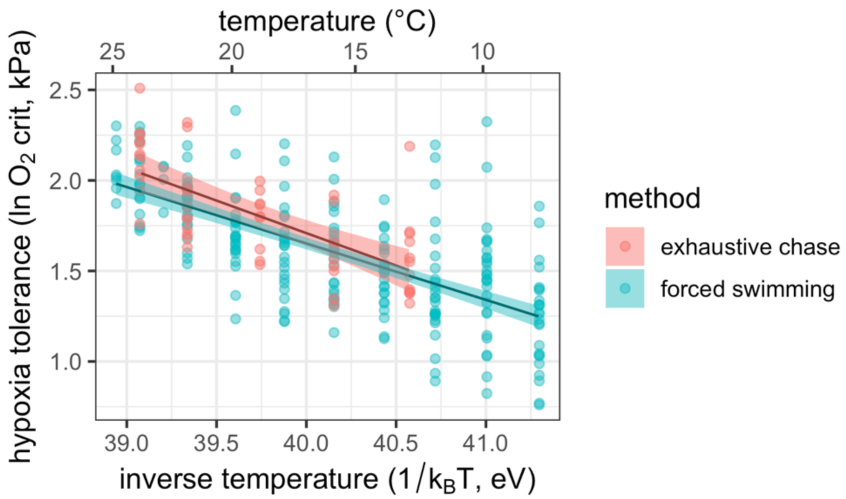

Supplementary Figure 4.

The method of maximum metabolic rate determination did not impact the temperature sensitivity of hypoxia tolerance in juvenile Chinook salmon. Data being compared (points) are fall-run smolts from Lo et al. (2022) (exhaustive chase) and Zillig et al. (2023a) (forced swimming) (n=285). Lines and ribbons show the fit ±95%CI of a multiple regression (MR) (R^2=0.48, $F_{3,281}$=88, p<0.001) with an interaction between inverse temperature and method of maximum metabolic rate determination. Importantly, the intercept and slope differences ±SE between exhaustive chase and forced swimming (-1.96±2.54 and 0.05±0.06, respectively) are not significant (t=-0.77, p=0.44 and t=0.75, p=0.46, respectively). Therefore, both methods yield statistically indistinguishable metabolic traits.

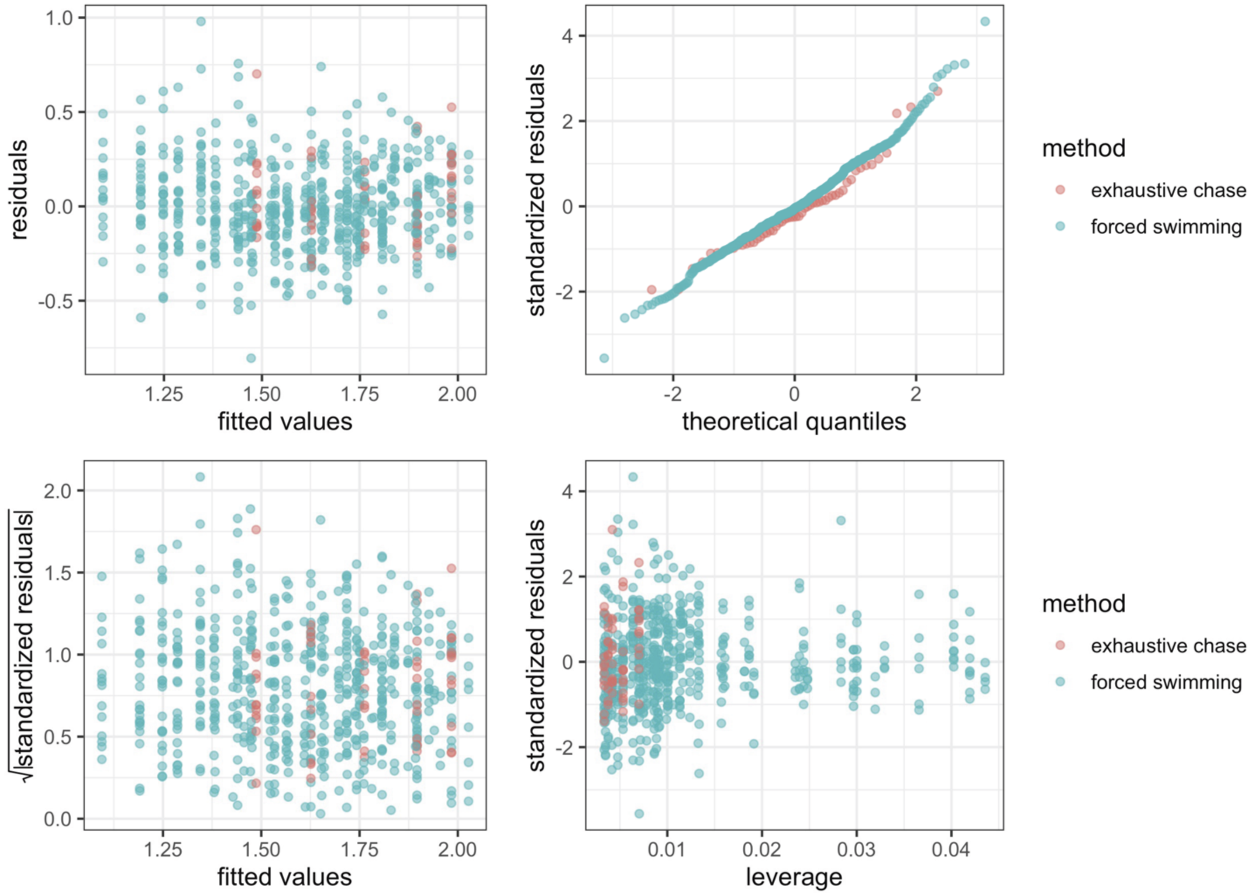

Supplementary Figure 5.

Method of maximum metabolic rate determination did not influence metabolic traits as quantified by the MR in our study. The residuals presented are those of the MR in Figure 1 (Supplementary Equation 2), which was used to produce metabolic traits in Table 1. Note that the residual patterns of exhaustive chase data match the residual patterns of forced swimming data.

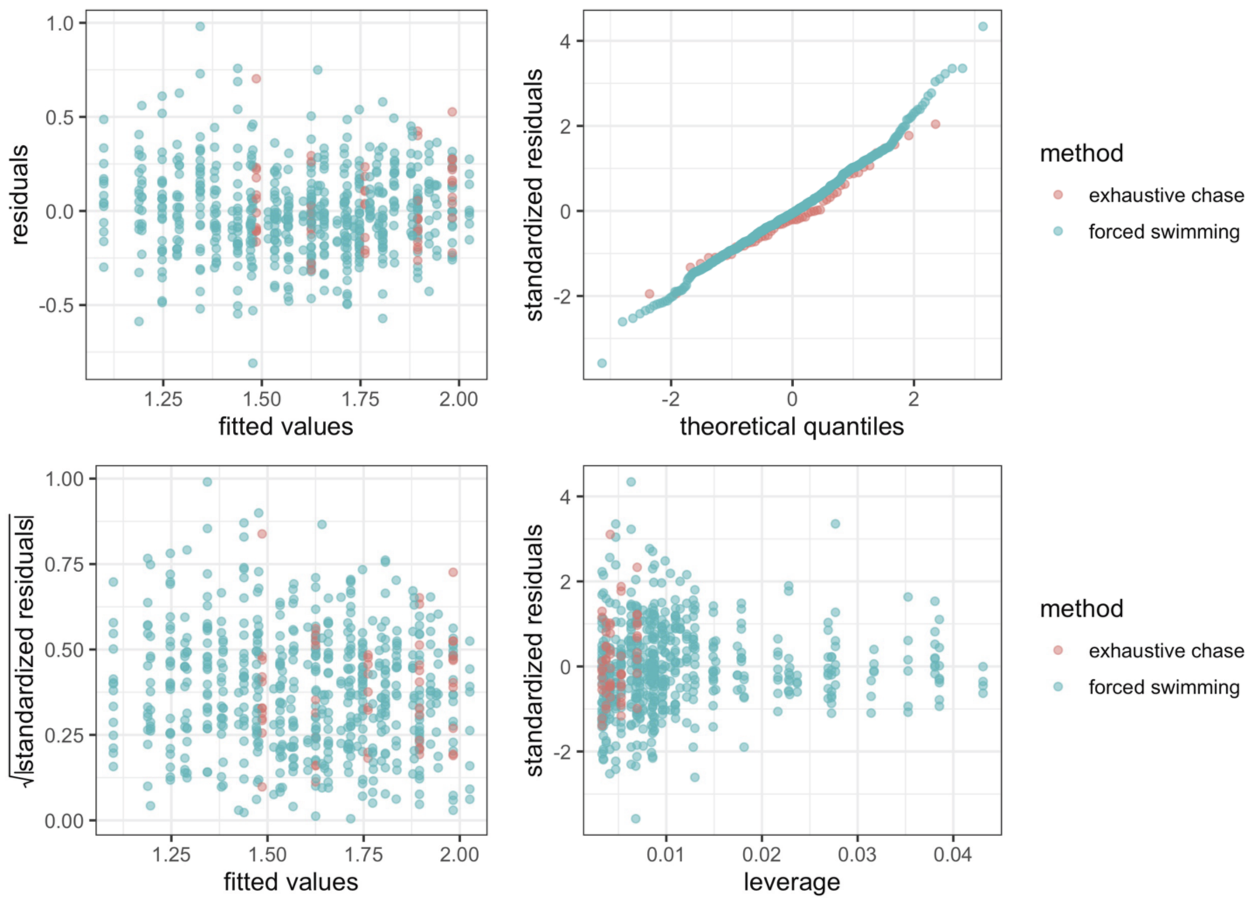

Supplementary Figure 6.

Method of maximum metabolic rate determination did not influence metabolic traits as quantified by the linear mixed effects regression (LMER) in our study. The residuals presented are those of the LMER in Figure 1 (Supplementary Equation 1), which was used to produce metabolic traits in Table 1. Note that the residual patterns of exhaustive chase data match the residual patterns of forced swimming data.

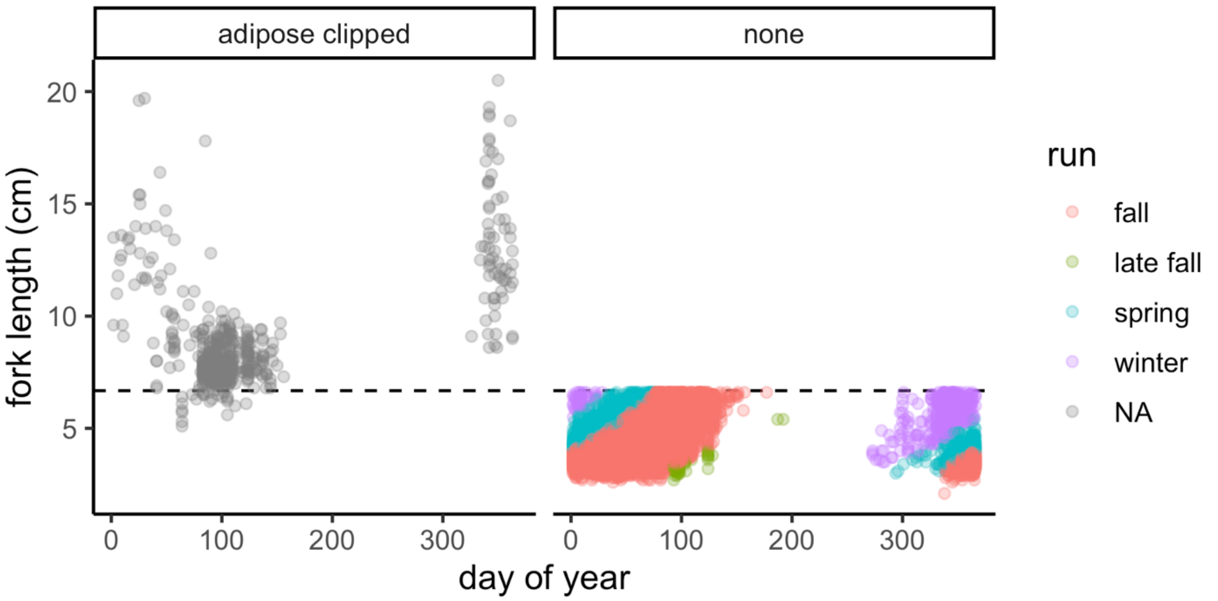

Supplementary Figure 7

Criterion for distinguishing wild fry in the rearing dataset. Size (fork length) of juveniles captured during sampling events vs. day of calendar year for (left panel) adipose-clipped (i.e., hatchery) and (right panel) unmarked (i.e., wild) juvenile Chinook salmon. The 0.05 length quantile of the adipose-clipped (i.e., hatchery) fish (6.7cm) is indicated by the horizontal dashed line. Right panel shows all unmarked juveniles below this size threshold, colored by likely population (i.e., run). If any unmarked Chinook salmon fry below this threshold were caught in a sampling event, that was classified as the presence of rearing fry. Plot produced from raw data available online (USFWS 2023).

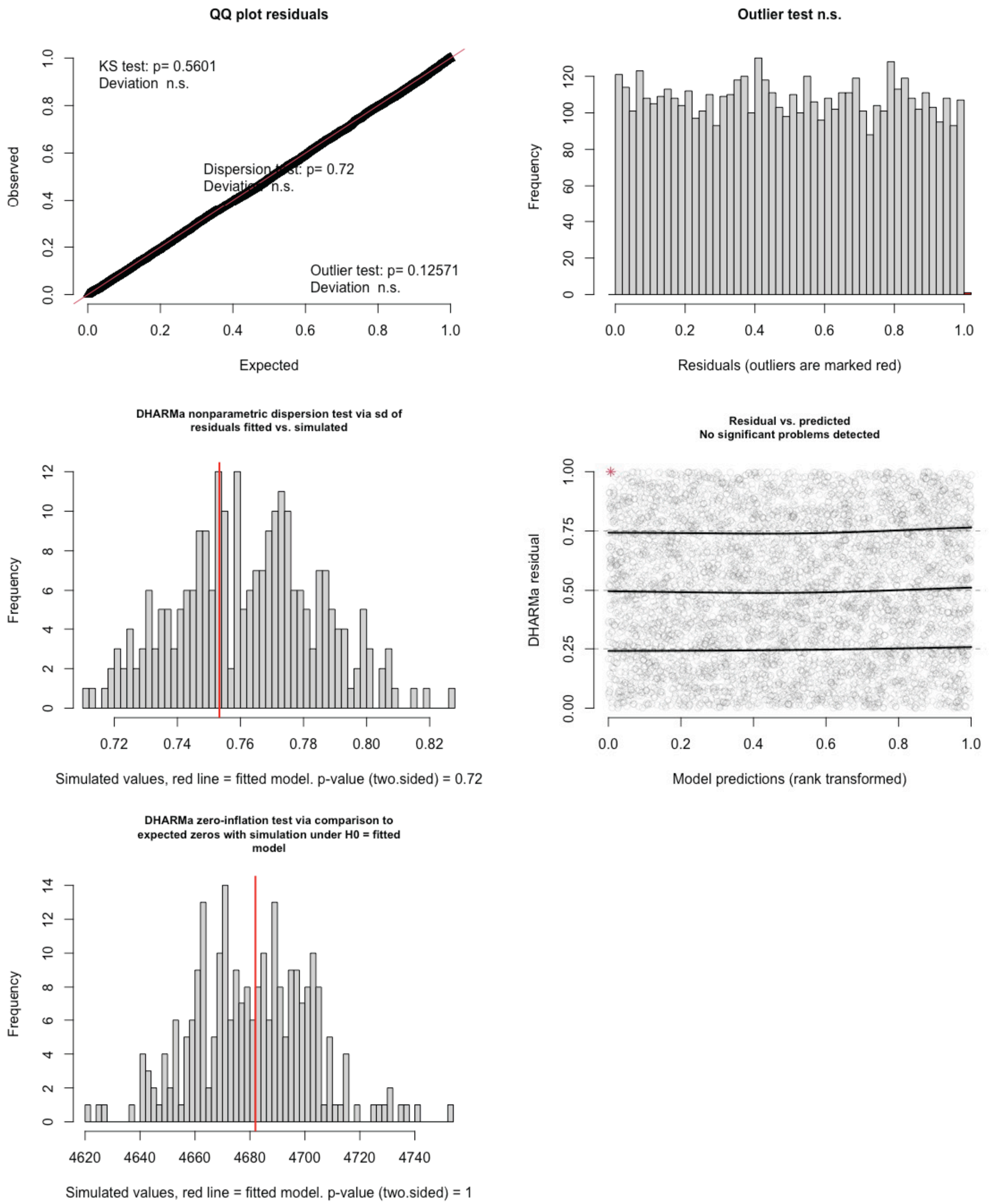

Supplementary Figure 8

Residual diagnostics of ϕ GAMM for Chinook salmon fry habitat use probability (Supplementary Equation 3).

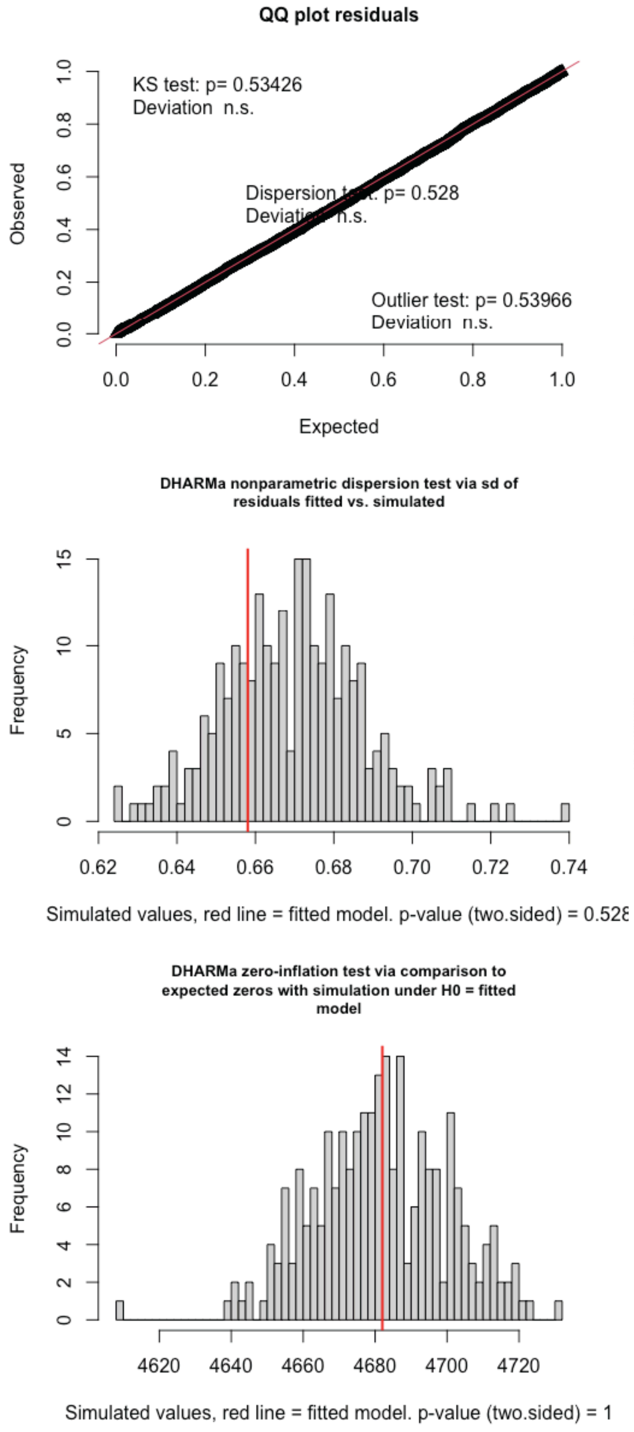

Supplementary Figure 9

Residual diagnostics of temperature GAMM for Chinook salmon fry habitat use probability (Supplementary Equation 12).

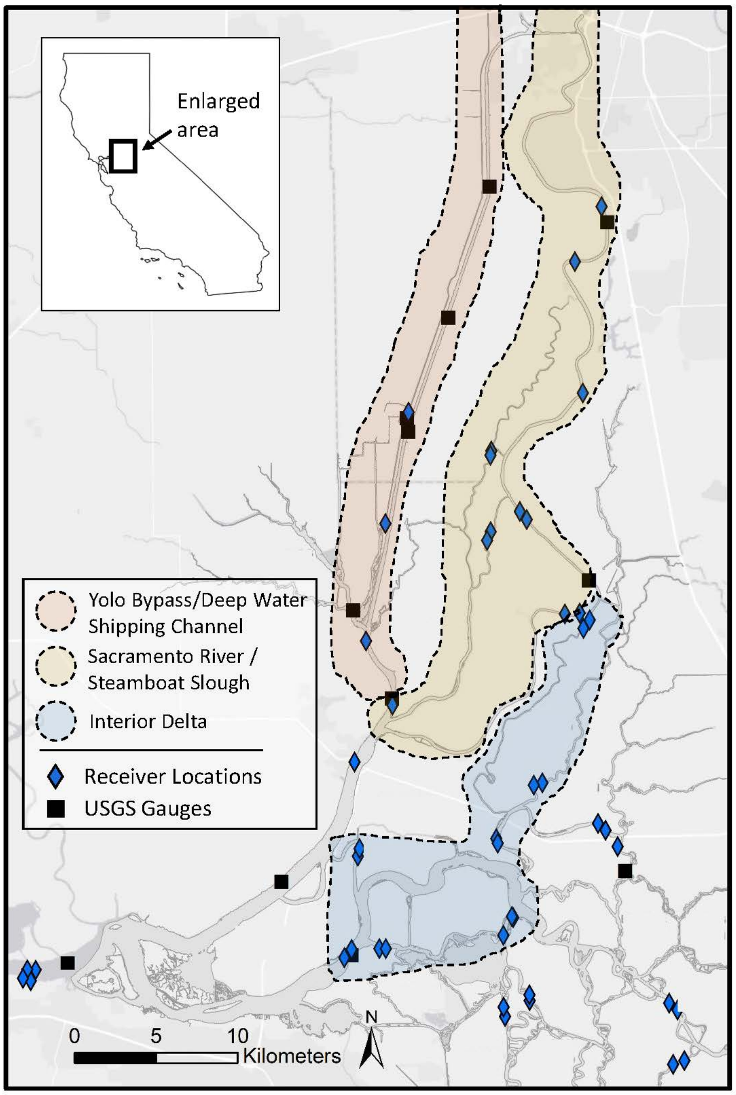

Supplementary Figure 10

Three general migratory routes taken by Chinook salmon smolts during the study period. Routes are outlined and color-coded. Blue diamonds show locations where Interagency Telemetry Advisory Group acoustic receivers listened for acoustically tagged Chinook salmon smolts during the study period (ITAG 2023). Black squares show US Geological Survey monitoring locations where temperature, dissolved O_2, and salinity, among other parameters, were recorded during the study period (USGS 2023).

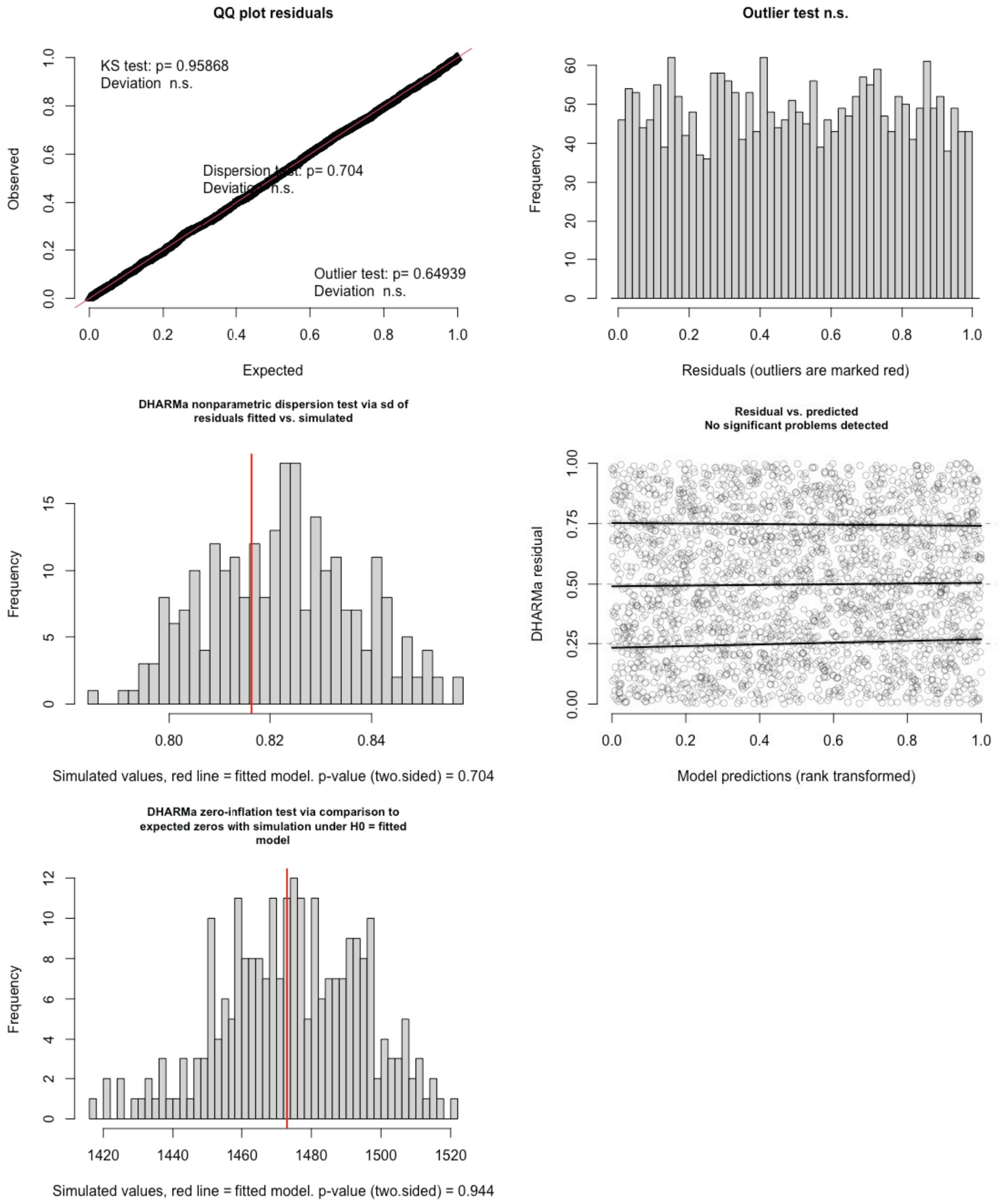

Supplementary Figure 11

Residual diagnostics of ϕ GAM for Chinook salmon smolt migration success probability (Supplementary Equation 4).

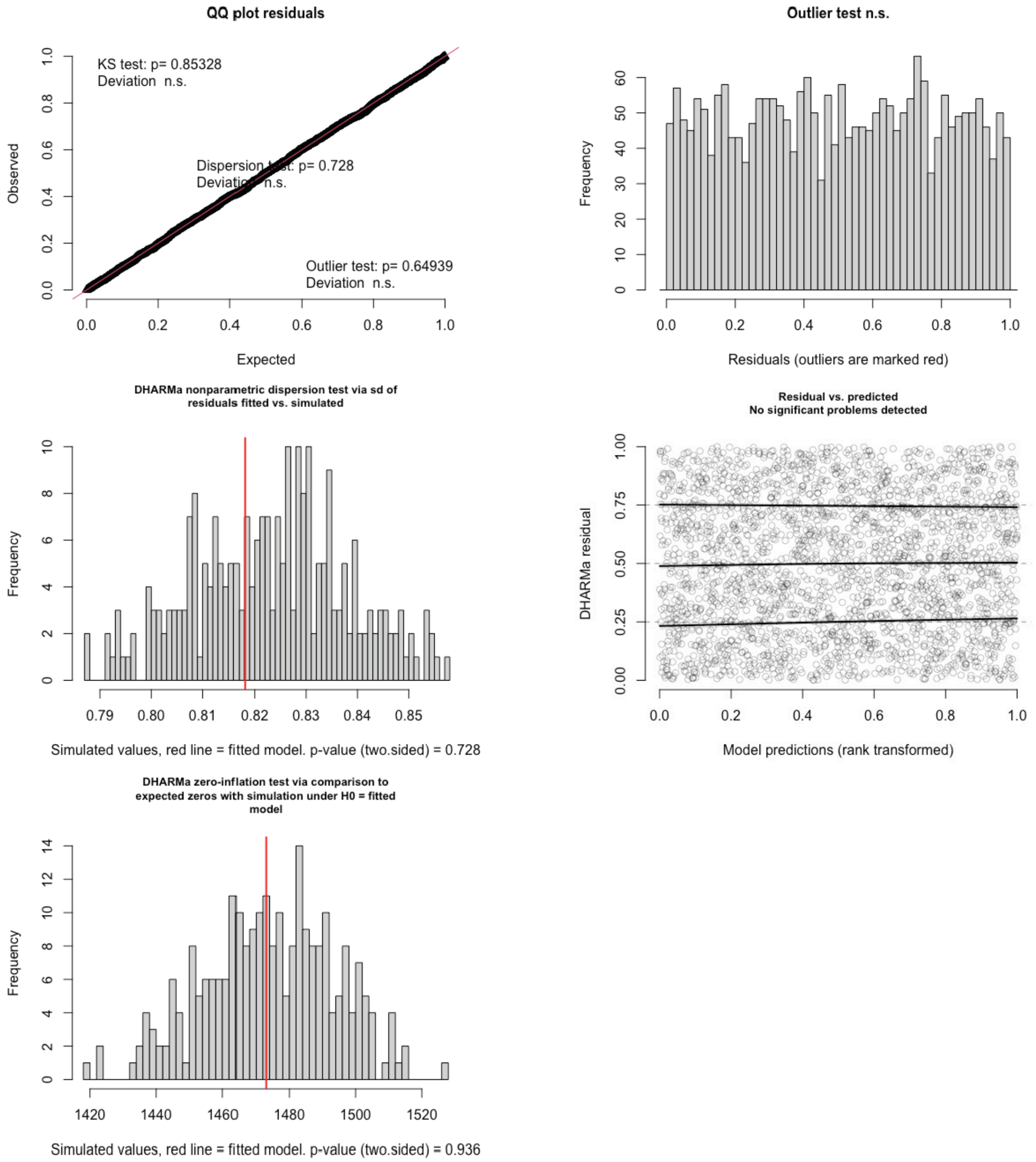

Supplementary Figure 12

Residual diagnostics of temperature GAM for Chinook salmon smolt migration success probability (Supplementary Equation 11).

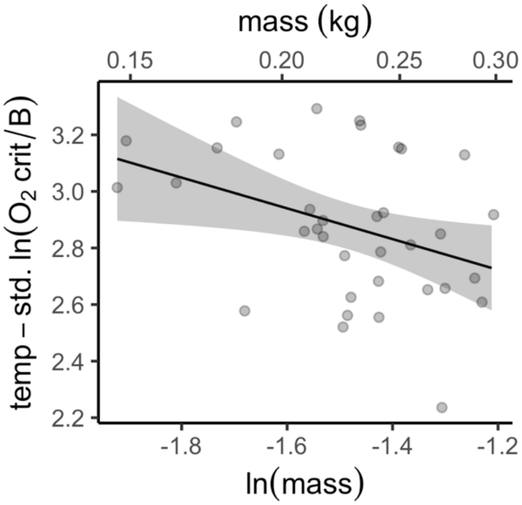

Supplementary Figure 13

Linear regression (LR) associating the natural logarithm of temperature-standardized, mass (B)-specific O_2crit with the natural logarithm of mass (kg) for adult largemouth bass. Points show data, while the line and ribbon respectively show the regression fit and 95% CI. Note that this fit was purely to mass-standardize the data and should therefore be interpreted within the bounds of this objective. The secondary x-axis (above) shows the corresponding mass in kg.

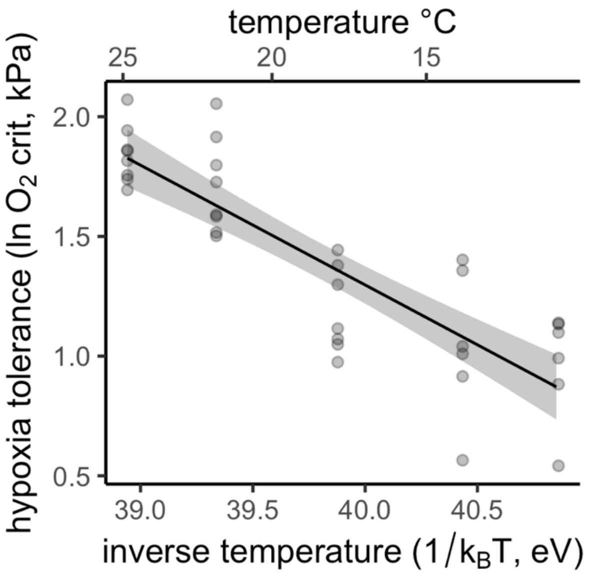

Supplementary Figure 14

Association between hypoxia tolerance and inverse temperature for largemouth bass. The intercept and slope of this association supplied the metabolic traits *A* and -*E*, respectively (Supplementary Table 3), which were used to parameterize the metabolic index (Equation 1) for largemouth bass. Points show data, while the line and ribbon respectively show the fit and 95% CI. The secondary x-axis (above) shows the corresponding temperature in °C.

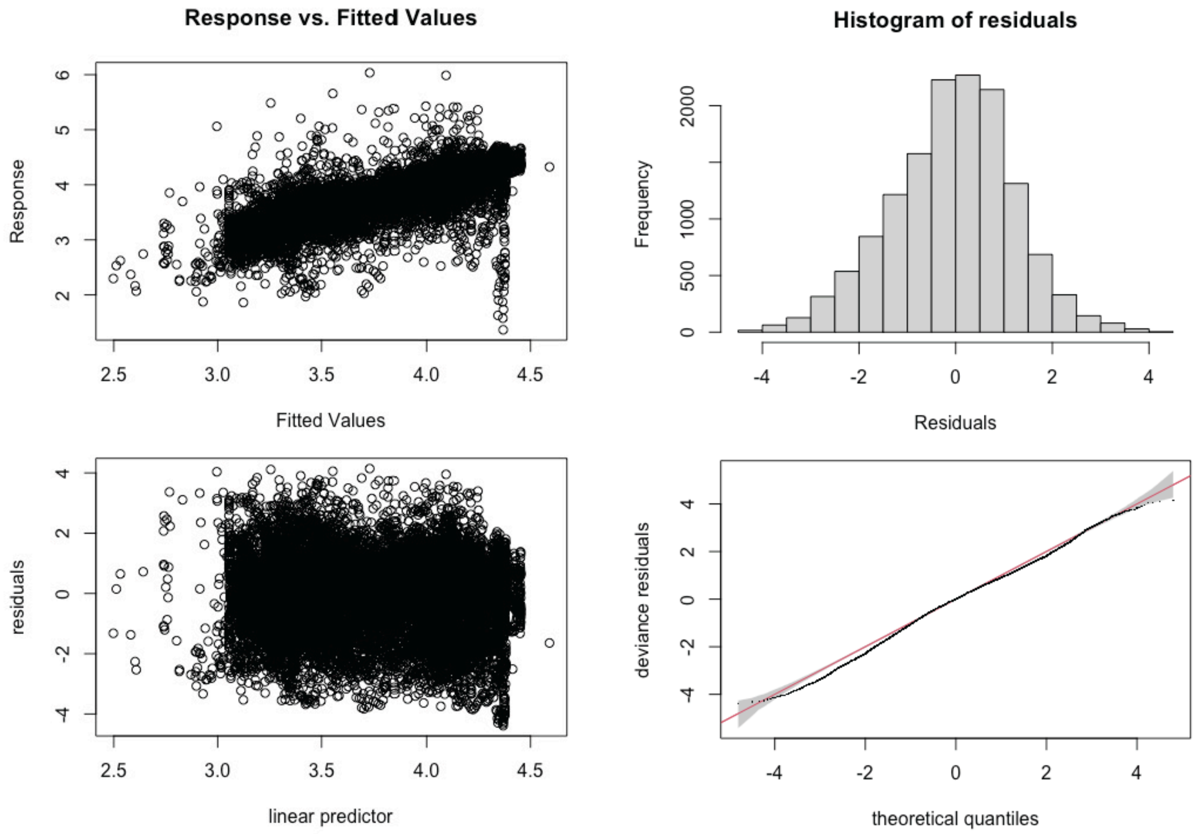

Supplementary Figure 15

Diagnostics of spatiotemporal ϕ GAM for Chinook salmon fry (Supplementary Equation 6).

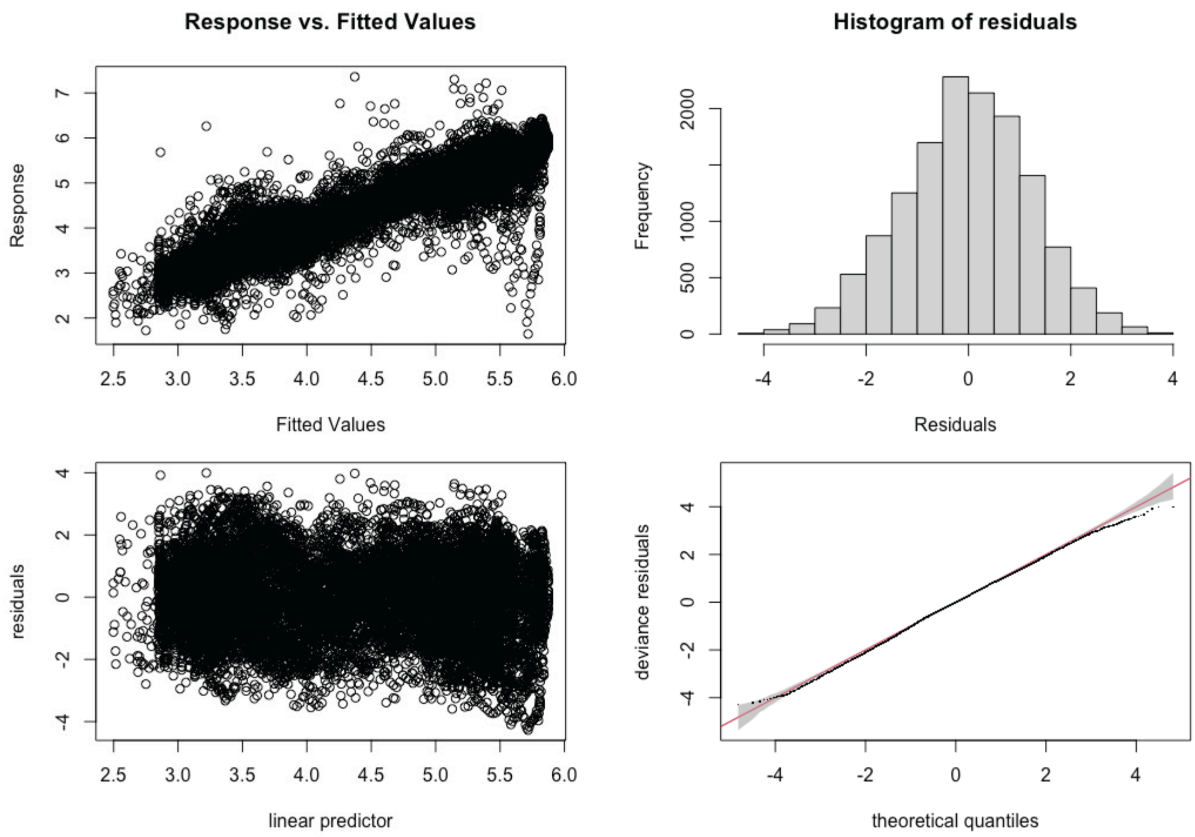

Supplementary Figure 16

Diagnostics of spatiotemporal ϕ GAM for Chinook salmon smolts (Supplementary Equation 5).

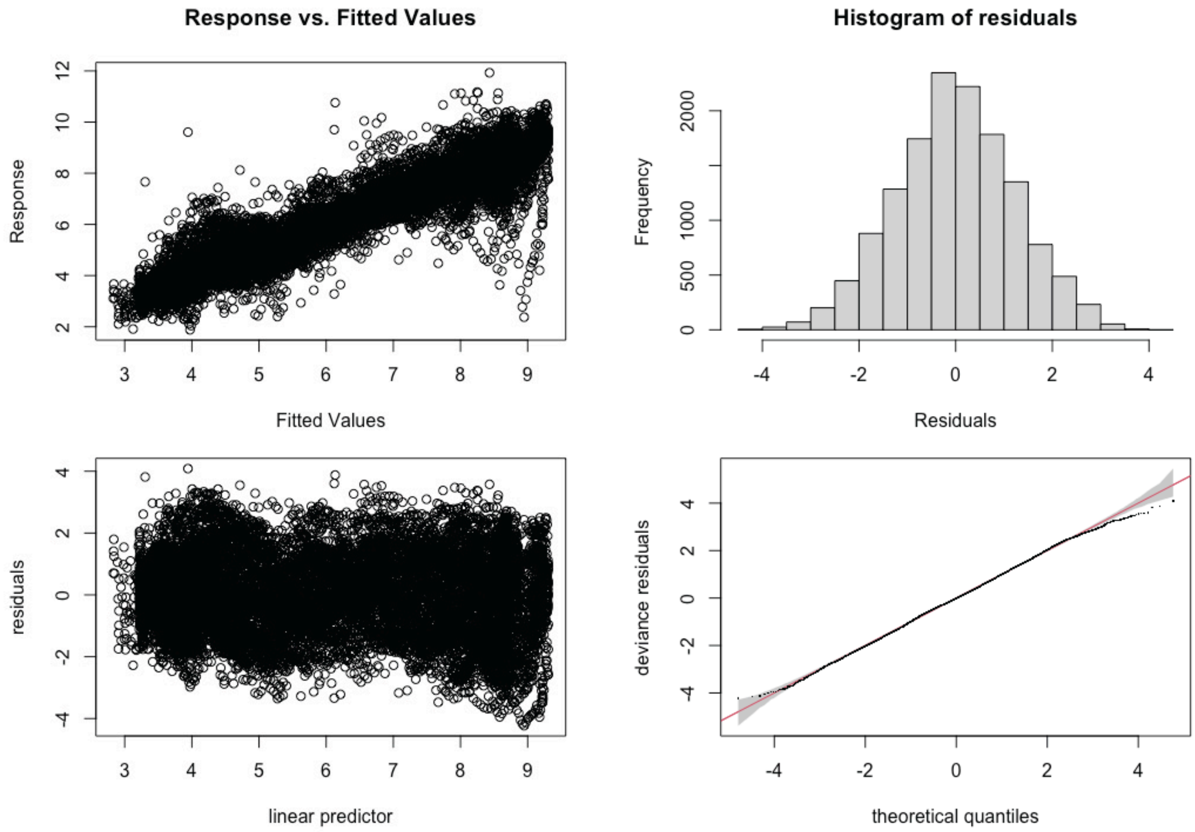

Supplementary Figure 17

Diagnostics of spatiotemporal ϕ GAM for largemouth bass (Supplementary Equation 8).

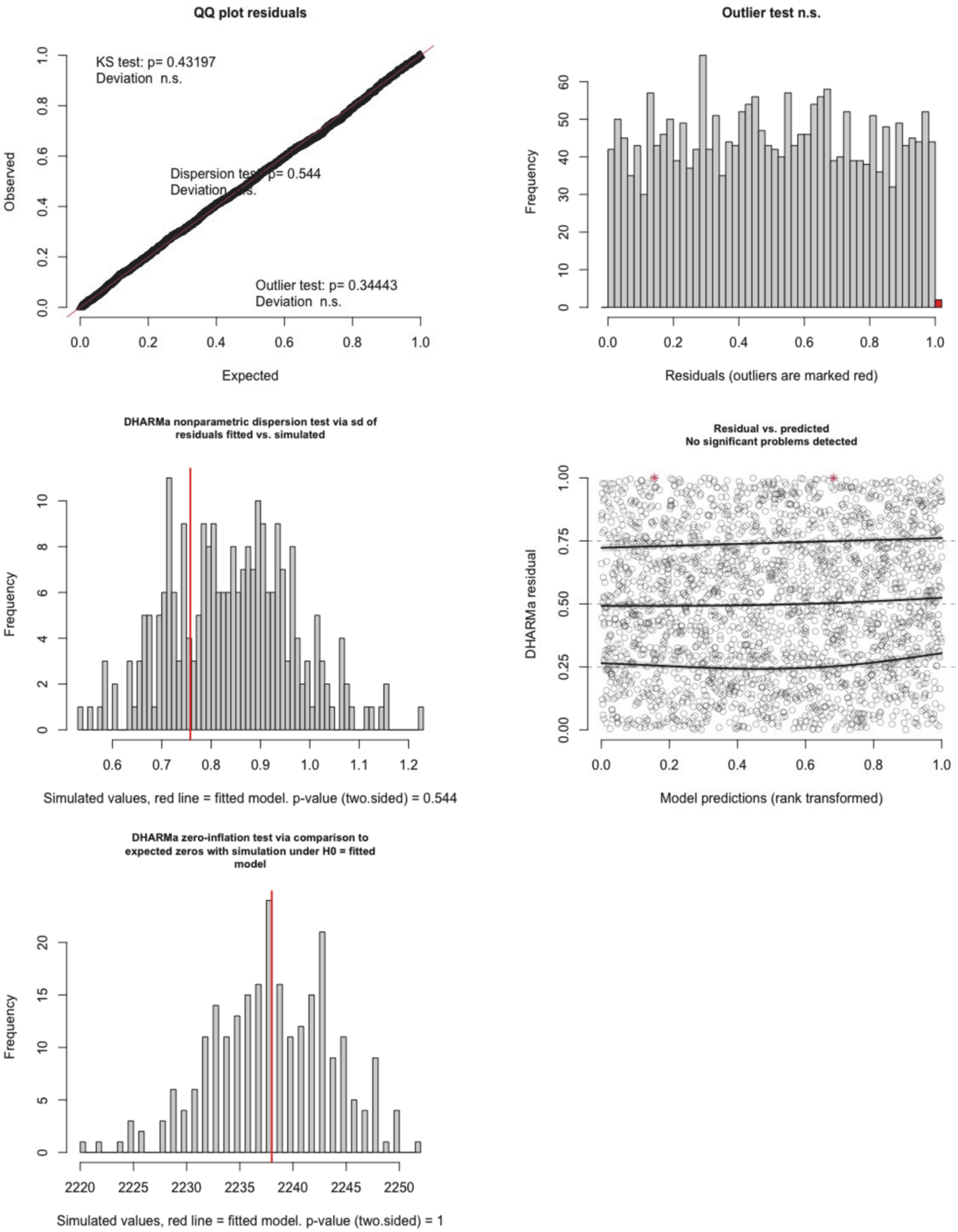

Supplementary Figure 18

Residual diagnostics of largemouth bass predation probability ϕ GAMM.

Supplementary Table 1

BIC results suggested a parsimony benefit for including population and lifestage in the model used to determine metabolic traits. Base model relates the natural logarithm of mass-standardized O_2crit with inverse temperature ($1/k_BT$, eV). ∆BIC refers to the difference in BIC compared to the base model, pop the population, and testT the test temperature.

model	df	BIC	∆BIC
base	3	10.37	0.00
base + pop	6	-9.39	-19.76
base + lifestage	4	13.97	3.61
base + pop + pop:testT	9	1.67	-8.70
base + pop + pop:testT + lifestage	10	-8.00	-18.37
base + lifestage + lifestage:testT	5	-3.79	-14.16
base + lifestage + lifestage:testT + pop	8	-38.71	-49.08
base + lifestage + lifestage:testT + pop + pop:testT	13	-21.24	-31.61

Supplementary Table 2

Temperature offered explanatory power over ϕ for fry rearing but not smolt migration. For fry (rearing), the base models (base) included a smooth term for ϕ or temperature (temp), a smooth term for flow, a parametric interaction between ϕ or temperature and flow, and a random intercept term for sampling location. For smolts (migration), the base models included smooth terms for ϕ or temperature (temp), flow, distance from release, and fish length, as well as a parametric interaction between ϕ and flow (interaction omitted from temperature model).

lifestage	model	df	BIC	ΔBIC
fry	ϕ base	24	3398.67	0.00
	temp base	23	2882.77	-515.90
smolt	ϕ base	20	2945.61	0.00
	temp base	19	2945.58	-0.03

Supplementary Table 3

Parameter values for *A*, (y-intercept) and *-E* (slope) for largemouth bass, determined from a LR (Supplementary Figure 14). Lower and upper 95% CIs are shown (p for all parameters <0.001).

model	parameter	value	low 95% CI	up 95% CI
LR	*A*	21.31	16.91	25.70
	-E	0.50	0.39	0.61

Supplementary Table 4

BIC results suggested a parsimony detriment when including distance from shore (ds) and time to night (ttn) as smooth terms (s[ ]) in the largemouth bass predation models, as well as a parsimony detriment when temperature replaced largemouth bass ϕ. The base model (base) included a smooth term for ϕ or temperature (temp), a parametric term for deployment duration, and random intercept terms for PER method and study site.

model	df	BIC	∆BIC
ϕ base	22	425.43	0.00
ϕ base + s[ds] + s[ttn]	28	439.86	14.43
temp base	22	426.83	1.40
temp base + s[ds] + s[ttn]	28	438.94	13.52

Supplementary Table 5

BIC results suggested no parsimony benefit for including acclimation temperature (accT) during mass standardization when determining the metabolic traits of juvenile Chinook salmon. The base model related the natural logarithm of temperature-standardized, mass-specific O_2crit with the natural logarithm of mass (kg). ∆BIC refers to the difference in BIC compared to the base model and accT the acclimation temperature.

model	df	BIC	∆BIC
base	3	154.99	0.00
base + accT	4	161.44	6.46
base + accT + ln(mass):accT	5	163.03	8.04

Supplementary Equation 1

LMER used to determine lifestage-specific metabolic traits averaged across populations (LMER in Table 1). In addition to inverse temperature (*invT*: $1/k_BT$, eV), lifestage (*life*: fry or smolt) and population (*pop*: fall, late-fall, winter, or spring) were included in this model. (*re[ ]* = random intercept, *:* = parametric interaction).

$$\ln(O_2crit) = invT + life + invT{:}life + re[pop]$$

Supplementary Equation 2

MR used to determine metabolic traits specific to lifestages of different populations (MR in Table 1).

$$\ln(O_2crit) = invT + life + invT{:}life + pop$$

Supplementary Equation 3

GAMM used to associate probability of habitat use by rearing fry (*rear*) with ϕ_f, flow ($m^{-3}s^{-1}$), and sampling location (*loc*: 15 locations where repeat sampling occurred). (*s[ ]* = smooth term).

$$rear = s[\phi_f] + s[flow] + s[\phi_f]{:}s[flow] + re[loc]$$

Supplementary Equation 4

GAM used to associate through-Delta migration success probability (*mig*) with ϕ_s, flow, migration distance (*migD*, km), and fish length (*length*, mm).

$$mig = s[\phi_s] + s[flow] + s[migD] + s[length] + s[\phi_s]{:}s[flow]$$

Supplementary Equation 5

GAM used to assess spatiotemporal patterns of ϕ_s in the Delta. Space was represented with latitude (*lat*) and longitude (*lon*), and time day of water year (*dowy*). (*ti[ ]* = smooth interaction).

$$\phi_s = s[lat, lon] + s[dowy] + ti[s[lat, lon], s[dowy]]$$

Supplementary Equation 6

GAM used to assess spatiotemporal patterns of ϕ_f in the Delta.

$$\phi_f = s[lat, lon] + s[dowy] + ti[s[lat, lon], s[dowy]]$$

Supplementary Equation 7

LR used to determine the metabolic traits of adult largemouth bass.

$$\ln(O_2crit) = invT$$

Supplementary Equation 8

GAM used to assess spatiotemporal patterns of ϕ_b in the Delta.

$$\phi_b = s[lat, lon] + s[dowy] + ti[s[lat, lon], s[dowy]]$$

Supplementary Equation 9

GAM used to associate predation probability of tethered smolts by largemouth bass (*pred*) with ϕ_b, PER deployment duration (*dur*, s), PER method (*method*: PER, sPER, or pPER), and study site (*site*: 30 locations where repeat sampling occurred).

$$pred = s[\phi_b] + dur + re[method] + re[site]$$

Supplementary Equation 10

GAM used to associate predation probability of tethered smolts by largemouth bass (*pred*) with *T* (°C) instead of ϕ_b.

$$pred = s[T] + dur + re[method] + re[site]$$

Supplementary Equation 11

GAM used to associate through-Delta migration success probability with temperature (*T*, C°) instead of ϕ_s.

$$mig = s[T] + s[flow] + s[migD] + s[length]$$

Supplementary Equation 12

GAMM used to associate probability of habitat use by rearing fry with temperature (*T*, C°) instead of ϕ_f.

$$rear = s[T] + s[flow] + s[T]: s[flow] + re[loc]$$

DataS1_R1.csv

Data for use with CodeS1_R2.R and CodeS2_R2.R. Empirical measurements used to determine metabolic traits that parameterized the metabolic index (ϕ) for juvenile Chinook salmon and largemouth bass. Each row represents measurements determined from individual fish. ref=reference (Zillig et al. 2023a, Lo et al. 2022, McInturf et al. 2022, or Zillig et al. 2023b), species=*Oncorhynchus tshawytscha* or *Micropterus salmoides*, lifestage=fry or smolt (if Chinook salmon), run=population of Chinook salmon (fall, late fall, winter, or spring), mass_kg=mass (kg), inv_T=inverse test temperature (eV), inv_accT=inverse acclimation temperature (eV), fas=factorial aerobic scope (unitless), pcrit_kPa=the oxygen required for standard metabolic rate (kPa). For raw data, see the relevant reference.

DataS2.csv

Data for use with CodeS2_R2.R. Beach seine sampling and environmental conditions used to determine Chinook salmon fry rearing probability in California's Sacramento-San Joaquin Delta (Delta). Each row represents a unique sampling event. ref=reference (USFWS 2023), date_time = time when sampling event was conducted in %Y-%m-%d %H:%M:%OS format with time zone Etc/GMT+8, catch_bin=0 (no fry caught) or 1 (fry caught), Location=name of sampling location where repeat measurements occurred, sac=tidally filtered average daily discharge of the Sacramento River into the Delta (m^3s^{-1}), WaterTemp=water temperature (°C), phi_f=fry-specific ϕ. For raw data, see the relevant reference.

DataS3.csv

Data for use with CodeS2_R2.R and CodeS3_R2.R. Acoustic telemetry and environmental conditions used to determine through-Delta migration success probability of Chinook salmon smolts. Each row represents an acoustically tagged juvenile Chinook salmon smolt that attempted to migrate through the Delta to the Pacific Ocean. ref_fish=telemetry reference (ITAG 2023), ref_hydro=environmental conditions reference (USGS 2023), ben_or_ds=0 (unsuccessful migration) or 1 (successful migration), free_time=time when smolt entered (or was estimated to enter) Delta in %Y-%m-%d %H:%M:%OS format with time zone Etc/GMT+8, release_river_km=migration distance (km), fish_length=fork length (mm), flow=tidally filtered average discharge of the Sacramento River into the Delta (m^3s^{-1}), temp=water temperature (°C), phi=smolt-specific ϕ. For raw data, see the relevant reference.

DataS4.csv

Data for use with CodeS3_R2.R. Environmental conditions used to determine spatiotemporal patterns of ϕ in the Delta. Each row represents a daily measurement at a given location. ref=reference (USGS 2023 or USFWS 2023), lat=latitude, lon=longitude, Date=date in %Y-%m-%d format, water_year_doy=day of water year (1-366), phi_f=fry-specific ϕ, phi_s=smolt-specific ϕ, and phi_b=largemouth bass-specific ϕ. For raw data, see the relevant reference.

DataS5.csv

Data for use with CodeS3_R2.R. Beach seine sampling of Chinook salmon fry in the Delta used as overlay in Supplementary Figure 2A. Each row represents a unique sampling event. As opposed to Data S2, these data include sampling from locations where fry were caught in fewer than 5% of sampling events over the entire 12-year sampling duration. ref=reference (USFWS

2023), lat=latitude, lon=longitude, Date=date in %Y-%m-%d format, and catch_bin=0 (no fry caught) or 1 (fry caught). For raw data, see the relevant reference.

DataS6.csv

Data for use with CodeS3_R2.R. Last detections of Chinook salmon smolts in the Delta aggregated by location and time used as overlay in Supplementary Figure 2B. Each row represents a unique monitoring location over a unique time range. ref=reference (ITAG 2023), lat=latitude, lon=longitude, bin_lab=date bin for plotting, and morts=number of unique fish with last known detections at a given location over a given time period. For raw data, see the relevant reference.

DataS7_R1.csv

Data for use with CodeS3_R2.R. Predation event recorder (PER) deployments and environmental conditions used to determine predation risk of juvenile Chinook salmon by largemouth bass. Each row represents a unique PER deployment. refs=references (Demetras et al. 2016, Michel et al. 2020a, Michel et al. 2020b, or Michel et al. 2023), per_method=PER type and time period (per_2014_2015, sper_2017, pper_2022), Site=name of sampling location where repeat measurements occurred, predation=0 (not predated) or 1 (predated by largemouth bass), Temp_C=water temperature (°C), DO_mgL=dissolved oxygen concentration (mgL^{-1}), dds_z=z-scored deployment duration (s), ds_z=z-scored median distance from shore (m), ttn_z=z-scored median time to night (min), and phi_b=largemouth bass-specific ϕ. For raw data, see the relevant reference.

CodeS1_R2.R

R code that determines metabolic traits of Chinook salmon and largemouth bass. Requires DataS1_R1.csv as an input. Produces Figure 1, Table 1, Supplementary Figure 1, Supplementary Figure 4, Supplementary Figure 5, Supplementary Figure 6, Supplementary Figure 13, Supplementary Figure 14, Supplementary Table 1, Supplementary Table 3, and Supplementary Table 5.

CodeS2_R2.R

R code that associates the aerobic scope of Chinook salmon with ecological fitness in the wild. Requires DataS1_R1.csv, DataS2.csv, and DataS3.csv as inputs. Produces Figure 2, Figure 3, Supplementary Figure 8, Supplementary Figure 9, Supplementary Figure 11, Supplementary Figure 12, and Supplementary Table 2.

CodeS3_R2.R

R code that determines the spatiotemporal patterns of aerobic scope, and assesses fitness detriments to Chinook salmon attributable to largemouth bass predation in an aerobic scope context. Requires DataS2.csv, DataS3.csv, DataS4.csv, DataS5.csv, DataS6.csv, and DataS7_R1.csv as inputs. Produces Figure 4, Figure 5, Supplementary Figure 2, Supplementary Figure 15, Supplementary Figure 16, Supplementary Figure 17, Supplementary Figure 18, and Supplementary Table 4.
